# Effect of Pre-Oxidation on Coagulation/Ceramic Membrane Treatment of Yangtze River Water

**DOI:** 10.3390/membranes11050369

**Published:** 2021-05-19

**Authors:** Shengji Xia, Xinran Zhang, Yuanchen Zhao, Fibor J. Tan, Pan Li, Yanling Liu

**Affiliations:** 1 Key Laboratory of Yangtze River Water Environment, Ministry of Education, Tongji University, Shanghai 200092, China; xiashengji@tongji.edu.cn (S.X.); zhangxinran197@126.com (X.Z.); zzzyccc@126.com (Y.Z.); lipan@tongji.edu.cn (P.L.); 2 Shanghai Institute of Pollution Control and Ecological Security, Shanghai 200092, China; 3 State Key Laboratory of Pollution Control and Resources Reuse, Tongji University, Shanghai 200092, China; 4 School of Civil, Environmental, and Geological Engineering, Mapua University, Manila 1002, Philippines; FJTan@mapua.edu.ph; 5 Yuchengco Innovation Center, Mapua University, Manila 1002, Philippines

**Keywords:** pre-oxidation, flat ceramic membrane, membrane fouling, microfiltration

## Abstract

The membrane separation process is being widely used in water treatment. It is very important to control membrane fouling in the process of water treatment. This study was conducted to evaluate the efficiency of a pre-oxidation-coagulation flat ceramic membrane filtration process using different oxidant types and dosages in water treatment and membrane fouling control. The results showed that under suitable concentration conditions, the effect on membrane fouling control of a NaClO pre-oxidation combined with a coagulation/ceramic membrane system was better than that of an O_3_ system. The oxidation process changed the structure of pollutants, reduced the pollution load and enhanced the coagulation process in a pre-oxidation-coagulation system as well. The influence of the oxidant on the filtration system was related to its oxidizability and other characteristics. NaClO and O_3_ performed more efficiently than KMnO_4_. NaClO was more conducive to the removal of DOC, and O_3_ was more conducive to the removal of UV_254_.

## 1. Introduction

Nowadays, water sources all over the world have been, and are being, seriously polluted. At the same time, high quality drinking water is required to obtain a better life. Therefore, advanced technologies are developed and applied gradually in water treatment plants to effectively remove the pollutants in water sources and meet the strict potable water regulations. Among these technologies, membrane filtration is one of the most promising, owing to its small footprint, reduced use of chemicals, selective separation, high efficiency, low operation cost and stable performance [1,2,3]. The membrane mainly includes an organic membrane and an inorganic membrane. Ceramic membranes are increasingly being considered as a cost-competitive and technically superior alternative to organic membranes for drinking water treatment [4,5]. Compared to organic membranes, inorganic membranes possess characteristics of high chemical, thermal and structural stability; inorganic membranes can be superior in resisting corrosive chemicals, microbial deterioration and the surface abrasion caused by coarse particle circulation [6,7].

However, membrane fouling due to the attachment of aquatic organic matter to the surface and/or inner structures of the membranes remains a major issue that is limiting the efficiency of ceramic membrane water treatment systems [8]. Various pre-treatment strategies, including adsorption using powdered activated carbon and chemical coagulation, have been employed to increase the efficiency and life expectancy of ceramic membrane processes [1]. Kennedy et al. reported that coagulation pre-treatment can improve membrane performance since particles become incorporated into flocs that are larger than the membrane pores; therefore, fewer particles have a chance of blocking the membrane pores [9,10]. Meyn and Leiknes compared three ceramic coagulant mixing schemes and their impact on the dissolved organic carbon removal, membrane fouling rate and residual coagulant concentrations [10,11]. Park et al. reported that the calculation of the proper coagulant dosage is an important factor controlling membrane fouling in the membrane processing of algal-rich water [12]. However, a coagulation pre-treatment alone is unable to control membrane fouling for a long-term operation and therefore additional or alternative methods are needed [13].

To optimize the coagulation/ceramic membrane process, oxidants have been put forward as a pre-treatment alternative for the ceramic UF membrane. Geno Lehman and Liu found that pre-ozonation was effective on the degradation of colloidal natural organic matter (NOM) [8,14]. Cheng et al. also reported that pre-ozonation with low doses efficiently alleviated the membrane fouling caused by humic acid (HA) and sodium alginate (SA) by converting the high-MW molecules into smaller ones that could pass through membrane pores and deteriorate the permeate quality [15]. Venne et al. demonstrated the potential of backwashes chemically enhanced with ozone to control the fouling of ceramic ultrafiltration membranes using cyanobacteria in a filtered surface water [16]. The objective of this study was to investigate the effect of pre-oxidation on the coagulation/ceramic membrane treatment of raw water from the Yangtze River.

The effects of the oxidant type and concentration on the anti-fouling property and selectivity of the coagulation/ceramic membrane combination process under pre-oxidation conditions were studied. The impact of pre-oxidation on membrane fouling was evaluated using the specific transmembrane pressure, and membrane selectivity was evaluated using the removal capacity of organic matter. Furthermore, the optimum species and concentration of oxidant were preliminarily determined. The results were expected to illustrate the feasibility of pre-oxidation on coagulation/ceramic membrane fouling control and the improvement of effluent quality.

## 2. Materials and Methods

### 2.1. Materials and Chemicals

The ceramic membrane used in this study was a flat, asymmetric ceramic membrane. The membrane material was α-Al_2_O_3_ with an average pore size of 100 nm. According to the manufacturer, the membrane had a narrow pore size distribution and, thus, a relatively high separation accuracy. The membrane module size was 250 mm × 80 mm × 6 mm and the effective filtration area of both sides was 400 cm^2^. Yangtze River water was used as a representative of natural raw water in China, which possessed a turbidity mainly ranging from 20 to 30 NTU during the experiment. The organic matter indexes of the raw water, i.e., the chemical oxygen demand (COD_Mn_), dissolved organic carbon (DOC) and ultraviolet absorbance (UV_254_) were 1.9–3.9 mg/L, 1.92–3.03 mg/L and 0.031–0.046 cm^−1^, respectively. The potassium permanganate (KMnO_4_), sodium hypochlorite (NaClO) and polyaluminum chloride (PAC) of analytical grade were purchased from Chemical Reagent Co., Ltd. (Shanghai, China). The ozone (O_3_) was obtained using an ozonizer (QS-10) from AQUA AIC (CHINA) Co., Ltd. (Shanghai, China).

### 2.2. Experimental Set-Up

**Figure 1:**
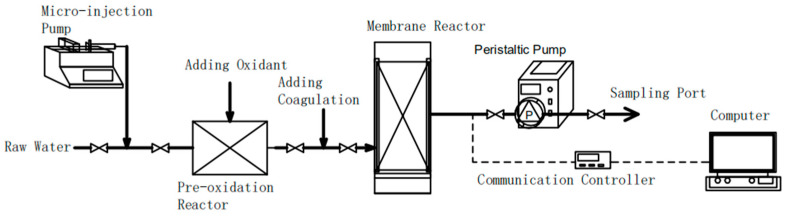
Schematic diagram of the experiment set-up.

All of the experiments were conducted using a laboratory-scale membrane set-up, as shown in Figure 1. It consisted of the following three parts: a mixing and dosing system, a membrane filtration section and a data recording system.

### 2.3. Operating Conditions

During the experiment, a metering pump continuously and quantitatively added coagulants to raw water from the Yangtze River. After rapid stirring, the colloidal substance was stabilized and allowed to flow into a membrane pool. The speed of mechanical agitator in the mixing tank was 350 r/min and the mixing time was about 2 min. An oxidant feeding unit was arranged before a metering pump. The solid-phase reactant was dissolved and added to an injection pump, and a gas-phase reactant was added using a water ejector. In particular, the O_3_ production for the pre-oxidation process was adjusted by controlling the O_3_ concentration (*C*_1_, mg/L) and flow rate (*Q*_1_, L/h) from the ozonizer. The O_3_ dosage (*C*_2_, mg/L) into raw water could be calculated using C_2_ = *C*_1_ × *Q*_1_/*Q*_2_, where *Q*_2_ (L/h) is the water flow rate of the system. A flat ceramic membrane was fixed at 6 cm below the liquid level of the membrane tank, and the out–in filtration mode was adopted. The outlet end of the membrane was connected to a constant current peristaltic pump by a pipe. The peristaltic pump provided filtration power, adjusted the filtration flux by tuning the speed of the peristaltic pump head, and switched between the filter and backwash modes by changing the rotation direction of the peristaltic pump head.

The ceramic membrane flux was constant at 120 L/(m^2^∙h) in this experiment. In order to keep the liquid level in the membrane tank constant, an overflow port was provided with a flow rate of 15 L/h. The pressure of the outlet pipe was measured and processed using a data recording unit. A pressure sensor was connected to the filter system, and the real-time data obtained was transmitted to a computer system using a communication controller. The concentrations of the three oxidants were selected according to those commonly adopted in previous studies [17,18,19] and set at three levels, respectively (Table 1). At the outlet of the pump, samples were collected and stored at a low temperature before the analysis.

### 2.4. Analysis and Calculation Methods

The turbidity and the COD_Mn_ value of the raw water were determined using a turbidimeter (2100N, HACH, Loveland, CO, USA) and an acid potassium permanganate titration, respectively. A total organic carbon analyzer (TOC-L CPH, SHIMADZU Corporation, Kyoto, Japan) was used to measure the DOC content in influent and effluent samples. Each water sample was filtered through a 0.45-micrometer membrane and adjusted to an approximate pH of 2–3 using HCl before the analysis. A UV–Vis scanning spectrophotometer (Hach DR6000 UV–visible, HACH, Loveland, CO, USA) was used to measure the absorbance of each sample at 254 nm. Fluorescence excitation–emission matrices (EEMs) were collected using a fluorescence spectrophotometer (Varian Cary Eclipse fluorescence spectrophotometer, Agilent, Palo Alto, CA, USA).

The membrane performance and the effect of backwash were monitored by logging the transmembrane pressure (TMP) development for constant flux operation and computed as follows:

TMP = P1 − P2(1)

where P1 and P2 are the osmotic pressures before and after transmembrane in kPa, respectively.

Based on the change in the transmembrane pressure before and after periodic backwashing, the total membrane fouling (TF) is divided into reversible membrane fouling (RF) and irreversible membrane fouling (IF) as follows:

TF = IF + RF(2)

During the test, to eliminate the influence of different initial transmembrane pressures (TMP_0_), the specific transmembrane pressure (TMP/TMP_0_) was introduced. The TF value of the nth cycle was represented by the difference in TMP/TMP_0_ between the final and the initial ratio of the cycle. The IF value was represented by the difference in TMP/TMP_0_ between the initial ratio of the first cycle and that of the n + 1th cycle, and the RF value was expressed as the difference in TMP/TMP_0_ between the end of the period and the initial ratio of the n + 1th period. The inflow and outflow were sampled to analyze the quality of the water, and the treatment’s effect was investigated by comparing the indexes of the raw water and treated water, including DOC, UV_254_ and three-dimensional fluorescence (EEMs). Excitation and emission slits were set to a 5 nanometer band-pass. The scanning rate of the xenon lamp used was 1200 nm/s and a 10 mm four-way quartz colorimetric dish was used as a sample pool. Surfer 8.0 and Origin Pro 9.0 software were used to map and analyze the data.

## 3. Results

### 3.1. Effect of Oxidants on the Specific Transmembrane Pressure Rate of Ceramic Membrane Filtration

**Figure 2:**
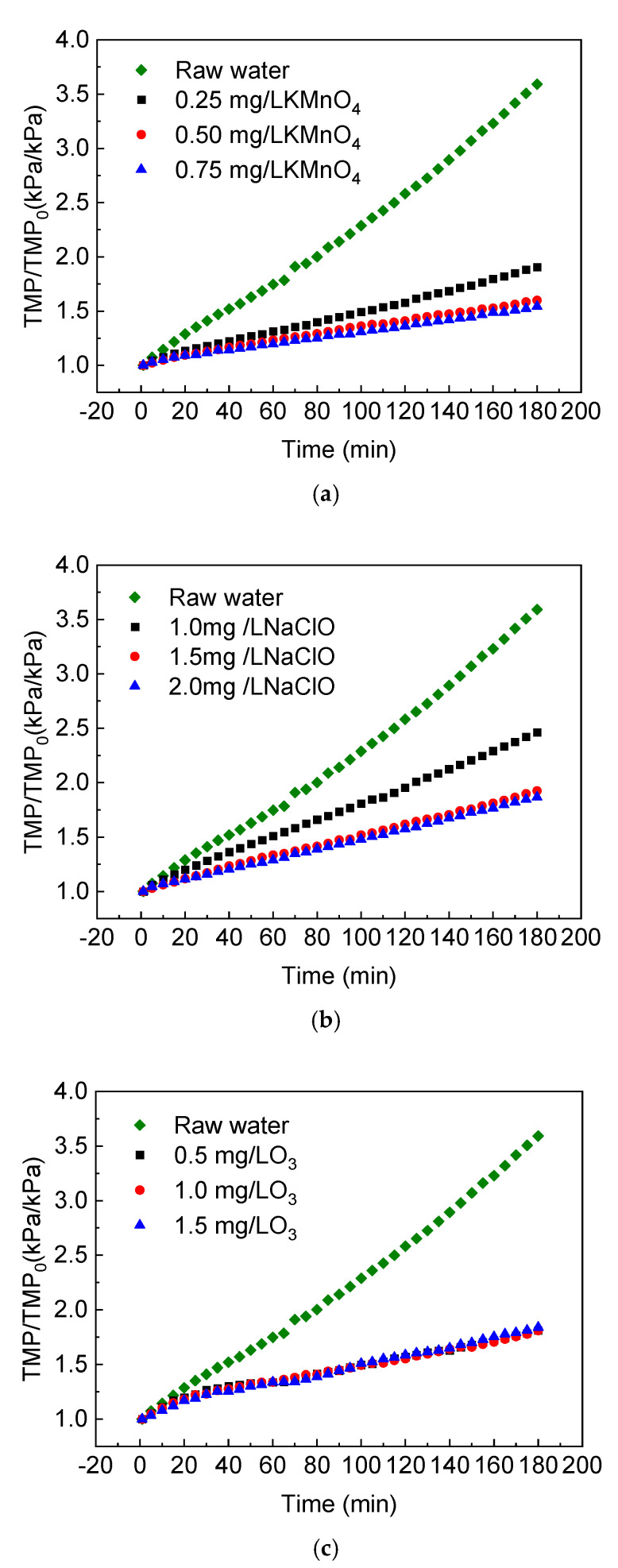
Effect of pre-oxidation on the specific transmembrane pressure of the ceramic membrane: (**a**) KMnO_4_; (**b**) NaClO; (**c**) O_3_.

The ceramic membrane flux was constant at 120 L/(m^2^∙h) during this experiment. The computer system recorded the pressure data in the pipeline during constant flux filtration in real time and plotted the rising curve of the membrane specific transmembrane pressure rate. Three different oxidants, including KMnO_4_, NaClO and O_3_, were used as pre-oxidants to study the effects of a pre-oxidation process on the specific transmembrane pressure rate of ceramic membrane filtration of the Yangtze River’s raw water. As shown in Figure 2, the pre-oxidation treatment greatly reduced the membrane fouling by natural water during continuous filtration, and the degree of mitigation was related to the type and concentration of the oxidants.

The ceramic membrane flux was constant at 120 L/(m^2^∙h) during this experiment. The computer system recorded the pressure data in the pipeline during a constant flux filtration in real time and plotted the rising curve of the membrane specific transmembrane pressure rate. Three different oxidants, including KMnO_4_, NaClO and O_3_, were used as pre-oxidants to study the effects of a pre-oxidation process on the specific transmembrane pressure. When the concentration of the pre-oxidant KMnO_4_ was 0.25 mg/L, the specific transmembrane pressure rate increased to 1.90 after a 180-min filtration and the fouling degree was only about 53% of the direct filtration of raw water. With the increase in the KMnO_4_ concentration, the increment of the specific transmembrane pressure rate decreased in turn during the same time. Within the range of 0–0.75 mg/L, when the KMnO_4_ concentration in water increased by 0.25 mg/L, the cumulative increase in the transmembrane pressure decreased by 1.69, 0.31 and 0.06, respectively. This showed that the degree of ceramic membrane fouling was negatively related to the concentration of oxidant in the KMnO_4_ pre-treatment and that adding KMnO_4_ had a good effect on the control of membrane fouling. However, with the increase in concentration, the contribution rate of unit concentration increments to membrane fouling control showed a downward trend. 

As shown in Figure 2b, the specific transmembrane pressure rate showed a decrease from 2.46 to 1.87 during the 180-min filtration, with an increasing NaClO dosage from 1.0 to 2.0 mg/L. Compared to raw water filtration, the relative proportion of fouling decreased from 68% to 52%. The degree of membrane fouling was negatively related to the oxidant concentration. The slope of the 1.5 and 2.0 mg/L NaClO curves were similar, which indicated that when the dosage was higher than 1.5 mg/L, increasing the concentration could no longer effectively reduce the degree of membrane fouling.

When comparing the three oxidants, the influences of both oxidant concentration and its oxidation power should be considered. Generally, KMnO_4_ and O_3_ were slightly better than NaClO in the control of membrane fouling, but the reasons were different. KMnO_4_ had a strong oxidation performance and its reaction with the pollutants in the raw water reduced the membrane fouling load and producing the by-product, manganese dioxide (MnO_2_). The MnO_2_ precipitated during the reaction generally existed in the form of fine colloidal particles with large surface areas and abundant hydroxyl functional groups on the surface [17,20]. It adsorbed hydrophilic organic compounds such as humic acid and further slowed down the membrane fouling to a certain extent. Adding O_3_ slowed the growth rate of ceramic membrane TMP mainly because of the unstable form of O_3_ in water. A hydroxyl radical with a strong electronic ability was formed by the interaction with the water matrix component, and NOM could be rapidly decomposed under the synergistic action of O_3_ and the hydroxyl radical [21]. In this experiment, the pH of the Yangtze River’s raw water was between 8 and 9. The reaction mentioned above was strengthened under alkaline conditions to mitigate membrane fouling. 

### 3.2. Effect of Oxidants on the Specific Transmembrane Pressure during Coagulation/Ceramic Membrane Filtration

**Figure 3:**
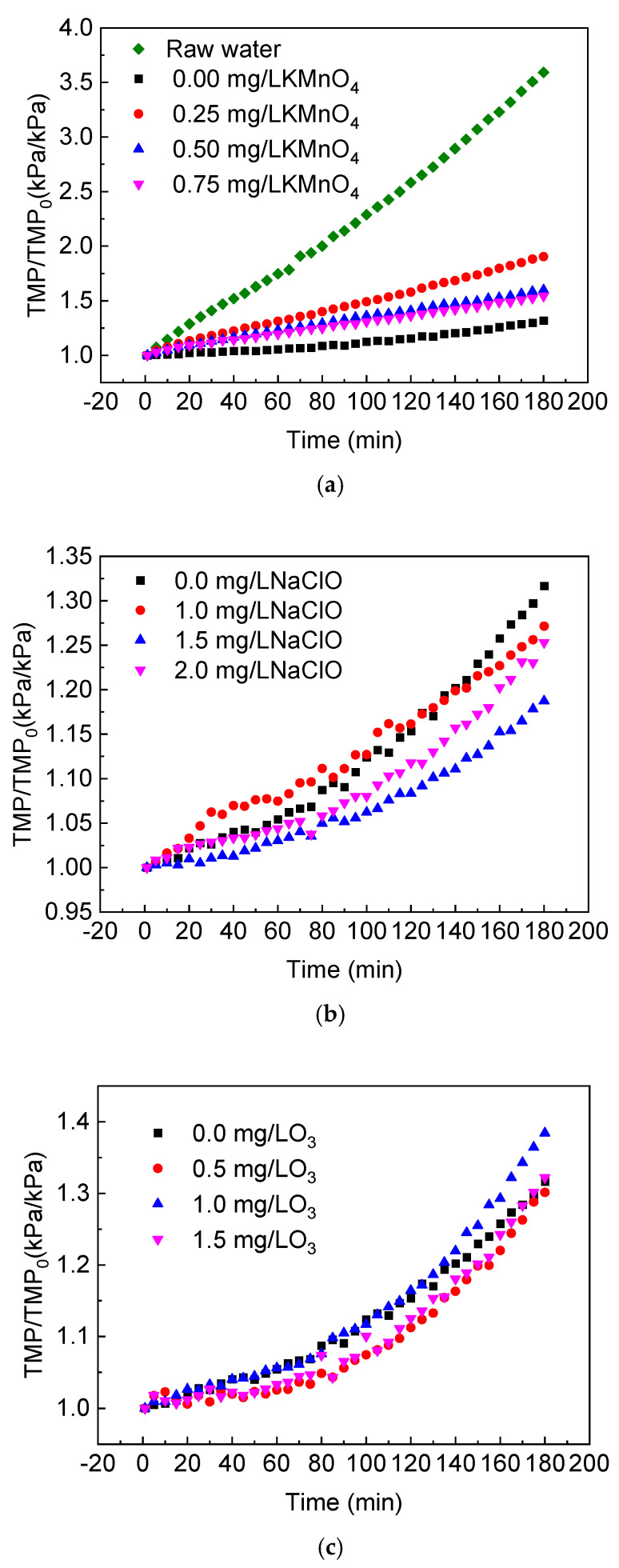
Effect of pre-oxidation on the specific transmembrane pressure of the coagulation/ceramic membrane process: (**a**) KMnO_4_; (**b**) NaClO; (**c**) O_3_ (the coagulant used was 2 mg/L PAC).

In this section, KMnO_4_, NaClO and O_3_ were used as pre-oxidants to study the effects of a pre-oxidation process on the change of transmembrane pressure over time in a coagulation/ceramic membrane combination process. The coagulant used was 2 mg/L polyaluminum chloride (PAC) and the experiment was performed using an online coagulation. A computer recorded the curve of the specific transmembrane pressure rate in each group and the results are presented in Figure 3. It can be seen that pre-oxidation combined with the pre-coagulation/ceramic membrane process could reduce the ceramic membrane fouling when treating natural raw water, but its effect was associated with the type and concentration of the oxidants.

Compared to an online coagulation/ceramic membrane filtration, adding KMnO_4_ could increase the degree of membrane fouling. At 0.25, 0.50 and 0.75 mg/L KMnO_4_ concentrations, the specific transmembrane pressure rate at the end of the 180-min filtration decreased in turn, but the results were higher than those of the experimental group without oxidants. The KMnO_4_ had a strong oxidation capacity and could oxidize large molecular hydrophobic organic matters into small molecular hydrophilic ones. The MnO_2_ produced by the reaction had a further absorption ability for organic matter; therefore, the organic load of the ceramic membrane decreased with the increase in the KMnO_4_ concentration. However, the addition of oxidants might also cause the transformation of natural organic matter from a granular state to a dissolved state and the adsorption ability of flocculation for dissolved organic matter was limited, which had an adverse effect on membrane fouling [22]. Secondly, fine MnO_2_ particles (generally 50–1000 nm [18]) that are insoluble in water could cover the ceramic membrane’s surface, which would change the structure of the cake layer and increase its resistance. This could be the reason why the degree of membrane fouling when using a KMnO_4_ pre-oxidation was greater than that of the coagulation/ceramic membrane system.

The effect of NaClO on membrane fouling in a coagulation/ceramic membrane combination system is related to the NaClO concentration and filtration time. At the beginning of filtration, the specific transmembrane pressure rate of 1.0 mg/L NaClO was greater than that of a coagulation/ceramic membrane system. After a 140-min filtration, the cumulative fouling of the latter exceeded that of the former, and this phenomenon might be related to the growth process of the cake layer structure. According to the order of the increase value of the specific transmembrane pressure rate in the coagulation/ceramic membrane system, the results were 1.0 mg/L > 2.0 mg/L > 3.0 mg/L. In other words, with the increase in the oxidant concentration, the optimization effect of the NaClO pre-oxidation on the combined process first increased and then decreased within a certain range. The optimum dosage was 1.5 mg/L. When using O_3_ as the pre-oxidant, the specific transmembrane pressure rate showed a trend of increasing steadily and then increasing rapidly. This was possibly due to the direct oxidation of organic matter by O_3_ in the early stages or the enhancement of the coagulation effect of PAC by modifying the functional groups of organic matter, which improved the anti-fouling performance of the coagulation filtration system. After the cake layer was formed on the membrane’s surface, it could, to a certain extent, block the effect of surplus oxidant on the inner and outer surface of the membrane, and thus accelerate the increase in the specific transmembrane pressure rate. As shown in Figure 3c, when O_3_ was added, the specific transmembrane pressure rate in the later filtration stage could exceed that without adding O_3_. This indicated that the cake layer formed with a pre-oxidant was denser, which was likely to cause more severe fouling in the cake layer stage. Therefore, when selecting O_3_ as a pre-oxidant, an appropriate filtration time should be selected. With the increase in the O_3_ concentration, membrane fouling increased first and then decreased, and the optimum O_3_ dosage was 0.5 mg/L.

Compared the three oxidants mentioned above, the KMnO_4_ pre-oxidation was not conducive to the long-term operation of the coagulation/ceramic membrane system from the perspective of membrane fouling control. Under suitable concentration conditions, the effect of the NaClO pre-oxidation combined with the coagulation/ceramic membrane system was better than that of the O_3_ system. In addition, it should be noted that the coagulant amount also had an influence on the membrane fouling rate of this combination process and the optimal PAC dosage could be specific to each kind of oxidant, which needs further investigation in future studies.

### 3.3. Removal of Organic Matter

**Figure 4:**
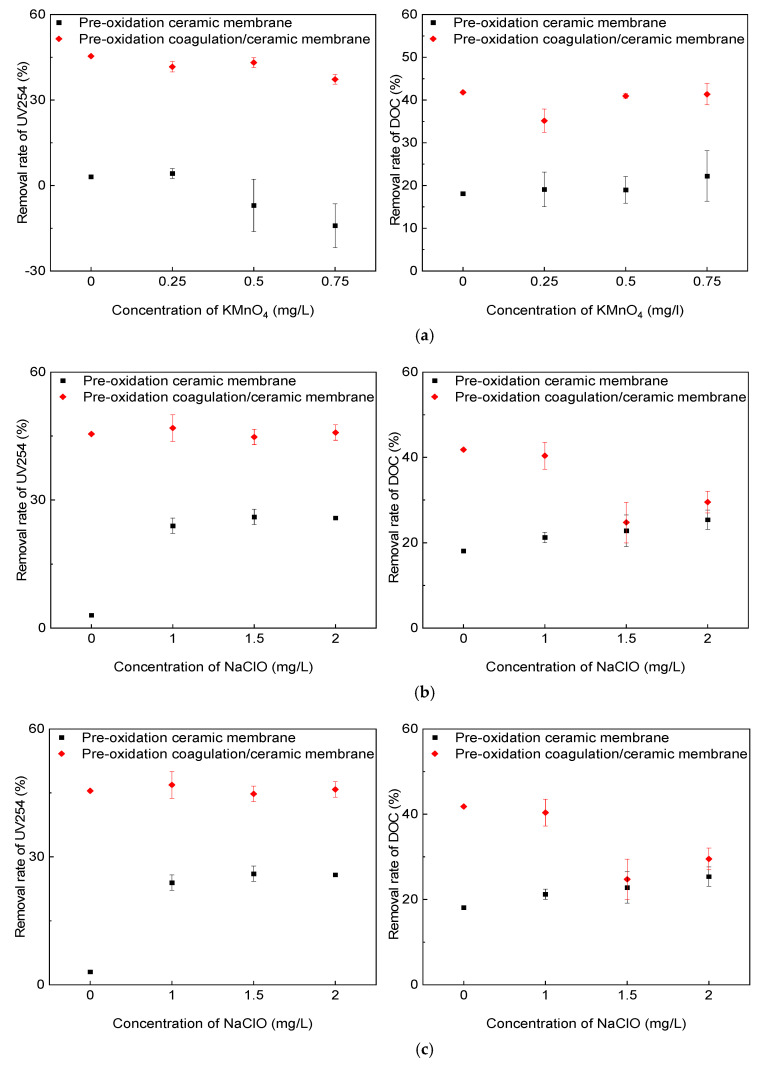
Effect of pre-oxidation on the removal rate of UV_254_ and DOC: (**a**) KMnO_4_; (**b**) NaClO; (**c**) O_3_.

The DOC and UV_254_ indexes in the influent and effluent in the direct membrane filtration and the coagulation/ceramic membrane process under the pre-oxidation of the three oxidants were measured. The removal rate was calculated to determine the effect of the oxidants on the removal capacity of organic matter. The coagulant was 2 mg/L PAC with an online coagulation condition in the combined process system. As shown in Figure 4, the removal rate of organic matter using the ceramic membrane was limited, which could be substantially enhanced by the addition of coagulants.

The influences of pre-oxidation using the three oxidants on the removal of organic matter were then evaluated. The results showed that the coagulation/ceramic membrane filtration process always exhibited better performance in removing organic matter than the direct filtration, regardless of the type and concentration of the oxidants. Comparing the three oxidants, O_3_ performed best in enhancing UV_254_ removal. The main reason for this is that O_3_ has a strong selectivity to unsaturated functional groups, such as the aromatic rings and double bonds of organic pollutants, while UV_254_ has a strong response to such functional groups. In the system combined with coagulation, the interaction between O_3_ and organic matter increased the number of acidic functional groups. It converted macromolecules into small molecules, reducing the electrostatic repulsion and steric resistance between flocs and organic components, thus promoting the effect of subsequent coagulation [23].

In terms of DOC removal by the coagulation/ceramic membrane system with pre-oxidation, the effect of the KMnO_4_ concentration on the DOC removal rate was found to be insignificant. However, increasing the dosage of NaClO or O_3_ decreased the DOC removal rate because excessive oxidants could destroy the structure of the cake layer and reduce its ability to intercept pollutants. In addition, the oxidation of undissolved organic matter could produce more small molecular fragments, making the effluent quality worse. 

### 3.4. EEM Fluorescence Spectrum

In this section, 3D fluorescence spectra were used to characterize the dissolved organic compounds in the influent and effluent under the pre-oxidation of O_3_ and NaClO, and the results were obtained using FRI (fluorescence regional integration). 

**Figure 5:**
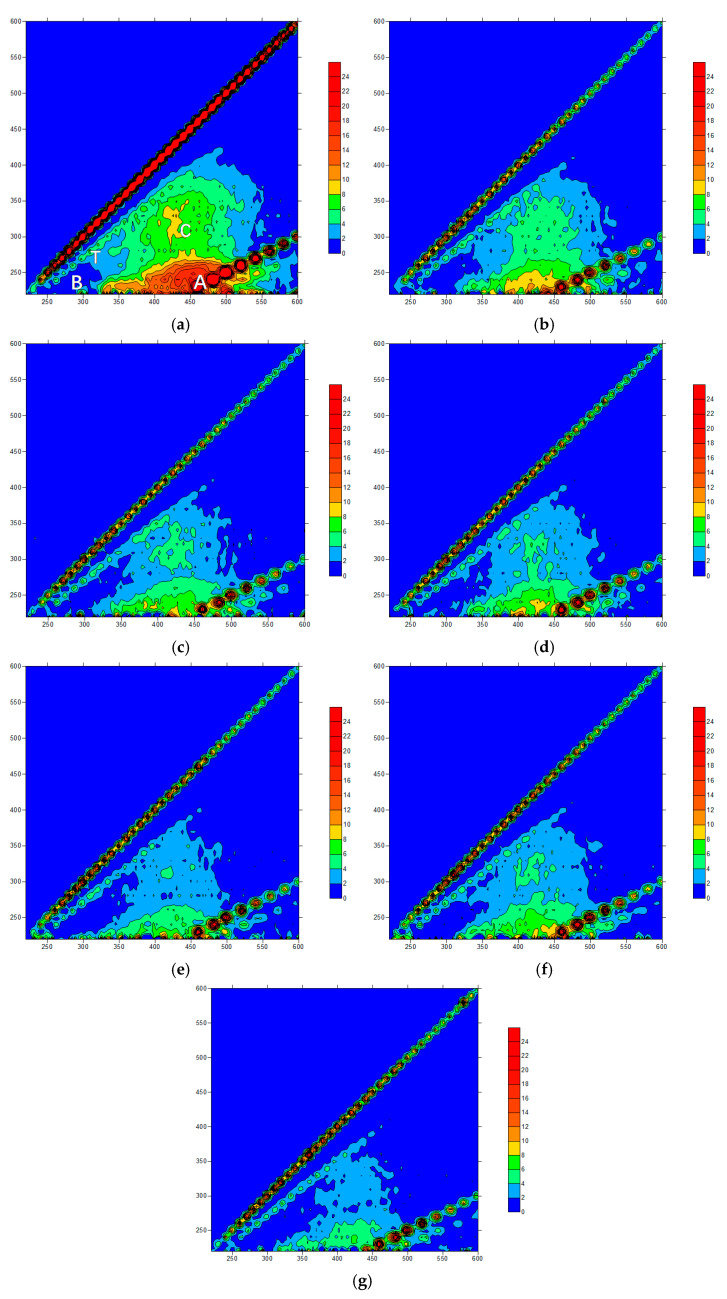
Fluorescence spectra of raw water and NaClO pre-oxidized effluent: (**a**) raw water; (**b**) 1.0 mg/L; (**c**) 1.0 mg/L + PAC; (**d**) 1.5 mg/L; (**e**) 1.5 mg/L + PAC; (**f**) 2.0 mg/L; (**g**) 2.0 mg/L + PAC.

**Figure 6:**
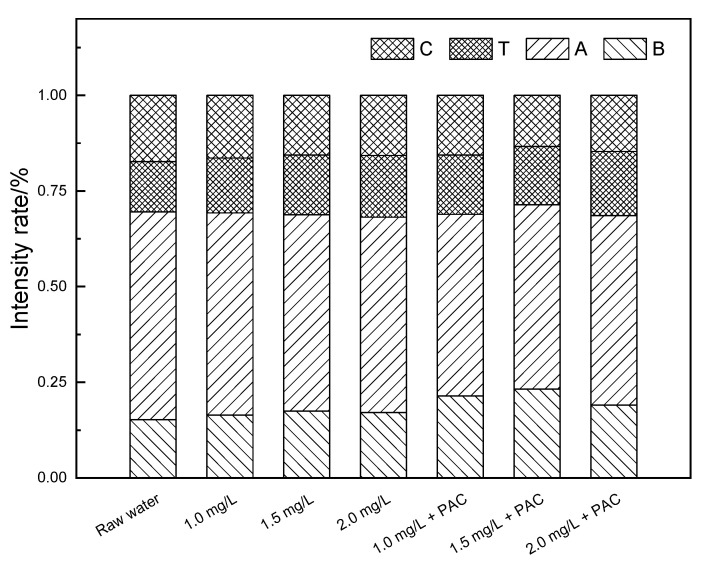
Proportion of the standard volume integral intensity of a NaClO pre-oxidation hydro fluorescent spectra.

As shown in Figure 5a, raw water had peaks in the T, B, A and C regions, which mainly correspond to soluble microbial by-product-like substances, proteins, fulvic acids and humic acids, respectively. The strong fluorescence response in region A indicated that fulvic acids were the main composition of the organic matter in the raw water. In the NaClO added ceramic membrane filtration system, the peak values of all the four regions decreased. With the increase in the oxidant concentration, the response value in each region decreased in turn and the response area decreased as well. This illustrated that the oxidation of NaClO could improve effluent quality and the removal ability of organic matter was related to the oxidant concentration. In the coagulation/ceramic membrane system, the effluent quality was greatly improved, suggesting that NaClO could strengthen the water purification efficiency of the coagulation/ceramic membrane system. Figure 6 showed that fulvic acids and humic acids were preferentially removed within the system, since the proportion of the standard volume integral in regions A and C reduced. In comparison, the relative strength ratios of the four regions remained approximately the same when the coagulant was not added, indicating that NaClO did not show selectivity to the oxidation ability of organic pollutants in the raw water from the Yangtze River. Therefore, coagulation could be considered as the main reason for the enhancement of a strong interception effect of the membrane on hydrophilic small molecular substances.

**Figure 7:**
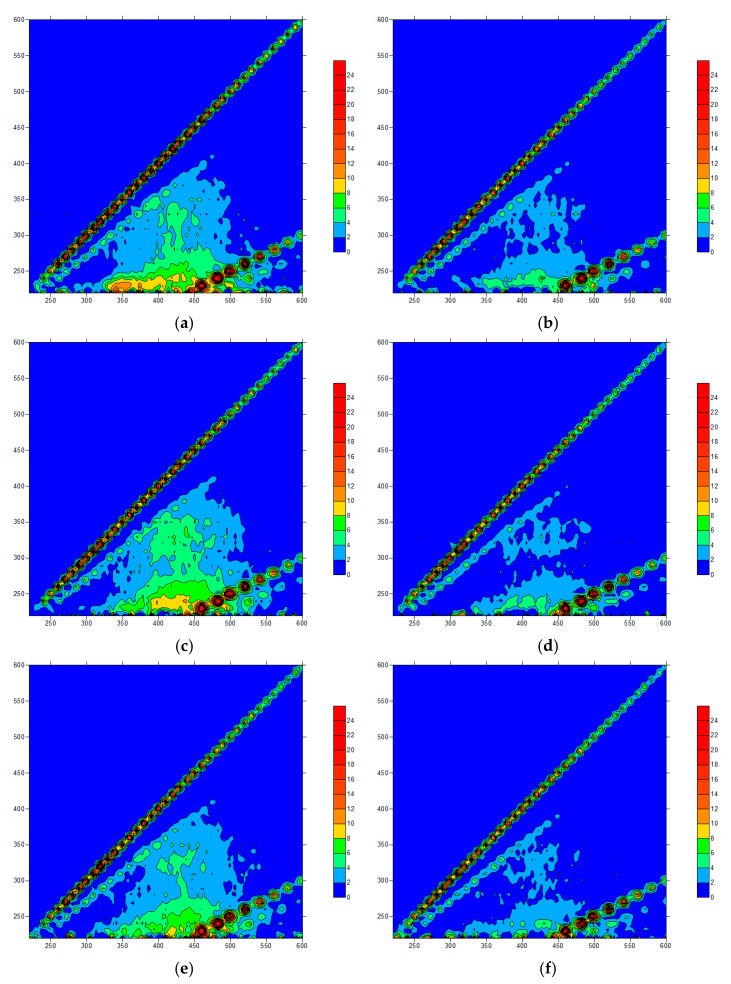
Fluorescence spectra of an O_3_ pre-oxidation effluent: (**a**) 0.5 mg/L; (**b**) 0.5 mg/L + PAC; (**c**) 1.0 mg/L; (**d**) 1.0 mg/L + PAC; (**e**) 1.5 mg/L; (**f**) 1.5 mg/L + PAC.

**Figure 8:**
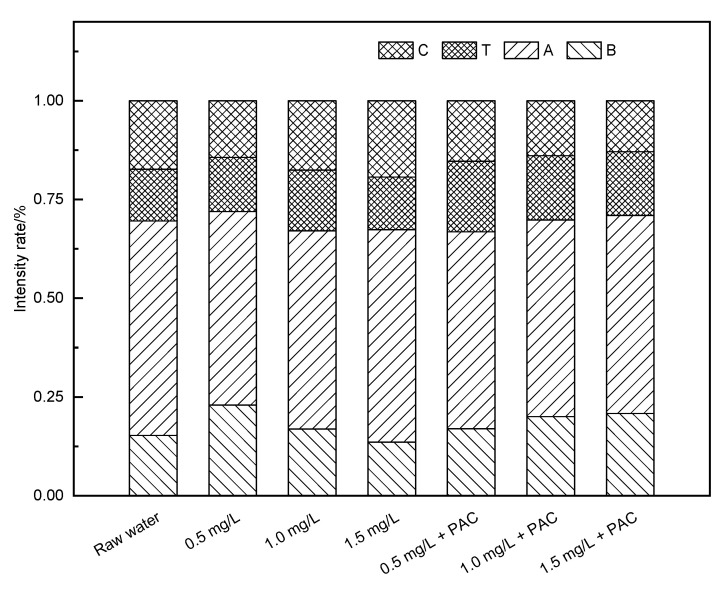
Proportion of the standard volume integral intensity of an O_3_ pre-oxidation hydro fluorescent spectra.

In the O_3_ added ceramic membrane filtration system (Figure 7 and Figure 8), when the O_3_ concentration was 0.5 mg/L, the response peaks in the A and C regions of the effluent samples were obviously weakened and the protein peak in region B showed no significant change. When the concentration increased to 1.0 mg/L, the protein substance decreased significantly, but the peak response in the C region increased a little. With the further increase in the O_3_ concentration, the response in each part of the fluorescence region was reduced. After coagulation treatment, the effluent quality of the system increased greatly, and the treatment’s effect was better than that of the NaClO. When the O_3_ concentration was low, the oxidation ability was weak, and the effect of oxidizing macromolecules was poor. Increasing the concentration made the O_3_ react incompletely with protein macromolecules and produce hydrophilic small molecular substances, increasing the response peak intensity in the A and C regions. When the O_3_ dosage was further increased, most of the organic matter was removed. In the O_3_ pre-oxidation combined with the coagulation/ceramic membrane system, the O_3_ and coagulation had a synergistic effect and thus the effluent quality was better than that of a non-coagulant system. The difference in the three-dimensional fluorescence spectrum of the effluent with different O_3_ concentrations was not significant, indicating that the combined process could be optimized just using O_3_ of a low concentration.

The intensity proportion in regions A and C were 54.3% and 17.3% in raw water, respectively. At a 0.5 mg/L concentration of O_3_, the intensity proportion in regions A and C were reduced to 49.8% and 15.3%, respectively. This suggested that the combined process had strong removal abilities for fulvic acids and humic acids, contributed by both O_3_ and PAC.

## 4. Conclusions

This study mainly investigated the characteristics of membrane fouling and the water purification effect of pre-oxidation on the filtration of raw water from the Yangtze River using a coagulation/ceramic membrane combination process. By changing the type and concentration of an oxidant, the mechanism of promoting or inhibiting the water purification characteristics of the combined system was analyzed. Through the experiments, the following conclusions were obtained:

(1) Both the pre-oxidation/ceramic membrane system and the pre-oxidation coagulation/ceramic membrane system could reduce the membrane fouling by natural water during continuous filtration. The latter was superior to the former in terms of membrane fouling control and water purification effect.

(2) There were two main reasons why oxidants could promote the water purification efficiency of the combined system. Firstly, it decomposed and transformed raw water pollutants through self-oxidation, and secondly, it changed the molecular structure of the organic matter and strengthened the coagulation effect. However, it must be noted that adding excessive oxidants had a negative effect on the filtration system. The excessive reaction destroyed the surface structure of the floc or cake layer, and thus reduced its pollution interception ability. On the other hand, a large number of small molecules formed by hydrolysis easily penetrated the membrane pores into the effluent.

(3) The hydrophilic molecules of humic acids and fulvic acids were preferentially removed in the pre-oxidation coagulation/ceramic membrane system, and NaClO and O_3_ were superior to KMnO_4_ as oxidants. This was related to the characteristics of the oxidant itself and the formation process of cake layers.

## Figures and Tables

**Table 1:** Concentrations of the oxidants.

Series	Oxidant	Concentration (mg/L)
1	KMnO_4_	0.25
		0.50
		0.75
2	NaClO	1.0
		1.5
		2.0
3	O_3_	0.5
		1.0
		1.5
